# Internet Addiction and Burnout in A Single Hospital: Is There Any Association?

**DOI:** 10.3390/ijerph18020615

**Published:** 2021-01-13

**Authors:** Gabor Toth, Krisztian Kapus, David Hesszenberger, Marietta Pohl, Gabor Kosa, Julianna Kiss, Gabriella Pusch, Eva Fejes, Antal Tibold, Gergely Feher

**Affiliations:** 1 Centre for Occupational Medicine, Medical School, University of Pécs, 7627 Pécs, Hungary; dtg1019@gmail.com (G.T.); kapusk@gmail.com (K.K.); pohlmarietta@bmvk.hu (M.P.); kosagabor@kosagabor.hu (G.K.); julianna.kiss@shl.hu (J.K.); efchengirl@gmail.com (E.F.); tibold.antal@pte.hu (A.T.); 2 Szent Rókus Hospital, 1085 Baja, Hungary; 3 Department of Laboratory Medicine, Medical School, University of Pecs, 7627 Pécs, Hungary; david.hesszenberger@aok.pte.hu; 4 Department of Neurology, Medical School, University of Pecs, 7627 Pécs, Hungary; puschgabriella74@gmail.com; 5 Hospital of Komlo, 7300 Komlo, Hungary; 6 Neurology Outpatient Clinic, EÜ-MED KFT, 7300 Komlo, Hungary

**Keywords:** internet addiction, burnout, healthcare professional, hospital

## Abstract

The extensive availability of the internet has led to the recognition of problematic internet use, the so-called Internet Addiction (IA), mostly involving adolescents. Burnout can lead to substance abuse or addictive behaviour (such as internet addiction) as a coping method. There are insufficient data about internet addiction and its possible association with burnout in adults, especially among healthcare workers. The aim of our present study was to focus on prevalence and the risk factors of internet addiction and its possible association with burnout among healthcare workers in a single hospital applying a questionnaire-based survey. In total, 49 doctors (10.1%), 198 nurses (40.9%), 123 medical assistant (25.4%), 73 other healthcare workers (15.1%), and 42 (1.7%) healthcare associated workers (cleaning, laundry, etc.) have completed our survey. In a multivariate analysis, IA was associated with age between 18 and 25 (OR: 2.6, *p* = 0.024), surfing on the internet >5 h daily (OR 25.583, *p* < 0.001), being single (OR: 4.275, *p* = 0.006), being childless (OR: 3.81, *p* = 0.011), working less than five years (OR 2.135, *p* = 0.048) and job type (being healthcare associated worker, OR: 2.907, *p* = 0.009). Illicit drug intake (OR 52.494, *p* < 0.001), and diabetes (OR: 4.122, *p* = 0.043) were also significantly associated with internet addiction. No association of burnout and IA could be found. A small but significant proportion of our healthcare workers suffered from IA, which was associated with substance abuse and diabetes in multivariate analysis. Our study also draws attention to the risk factors of IA such as younger age, family status, working type and working hours internet use. The possible association of burnout and IA merits further investigation.

## 1. Introduction

The widespread use of the internet has dramatically changed our lives in the 21st century. Although this technology has improved many aspects of our lives and it is now an essential part of the everyday routine including work, private life and social functioning, many studies have reported the misuse of the internet (problematic internet use, internet addiction; IA) as summarized in a recent meta-analysis [1]. The individual suffering from internet addiction may not be aware of it and it remains unrecognized by their relatives, friends and colleagues [2]. IA may be classified as a compulsive-impulsive spectrum disorder based on symptomatology, but it has been under considerable research, and not included in the recently published 5th edition of the Diagnostic and Statistical Manual DSM-V [3,4]. 

IA seems to have several risk factors such as younger age at the start of the internet use, being male, daily time interval and low socioeconomic status [3,4]. Psychosocial factors such as low self-concept and lack of family support are also associated with problematic internet use [5,6]. Problematic internet use seems to be associated with medical conditions such as anxiety, depression, drug abuse and malnutrition [7,8]. Interestingly, intrapersonal variables were statistically shown to have larger effects on IA than interpersonal variables according to a recent meta-analysis [9]. IA is mainly studied in adolescents, but it may also occur among adults and can be associated with greater mental symptom burden and fatigue [10].

Burnout is an increasingly prevalent syndrome resulting from situational, personal and professional stress (mainly caused by workplace stress) and can be characterized by emotional exhaustion, depersonalization, and reduced personal accomplishment [11]. The most vulnerable people are those working in human services, especially healthcare professionals, but it affects many other occupations. It has a great impact both on the individual and on the society as it may lead to undesirable consequences such as emotional depletion, loss of energy, dehumanization, detachment from work, feeling of inadequacy, reduced productivity and coping skills [12].

Although it is categorized as an occupational phenomenon and not as a medical condition, it seems to be strongly associated with somatic issues such as cardiovascular disorders or chronic pain syndromes [12]. Furthermore, it strongly correlates with mental disorders such as depression and insomnia, and a recent publication labelled burnout as a possible underlying cause of substance abuse and addiction [12,13].

Based on the above-mentioned findings, burnout can lead to substance abuse or addiction (such as internet addiction) as a coping method for anxiety symptoms, job dissatisfaction and negative emotions. Excessive internet use can lead to later school burnout and vice versa based on a recent study in adolescents [14]. 

However, only limited data are available about the prevalence and risk factors of the internet addiction among adults especially among healthcare professionals. Moreover, the association of burnout and internet addiction is not well studied.

The aim of our study was (a) to detect the prevalence of internet addiction, (b) to identify its risk factors and (c) to analyze its possible association with burnout among healthcare workers in a single hospital, by applying a questionnaire-based survey.

## 2. Materials and Methods

### 2.1. Participants

This study was conducted between January and August 2020 in Szent Rókus Hospital, Baja, Hungary.

The study was approved by the Ethical Committee of Szent Rókus Hospital, Baja, Hungary.

Inclusion criteria for the participants were working with human services, being between 18 and 65 years of age and being employed at the time of the study regardless of the type of employment (public servant, sub-contractor, etc.). 

Exclusion criteria were being under 18 or over 65 years of age, taking a leave of absence or refusal to participate in the study.

Demographic criteria included age, gender, marital status, number of children, type of work, years spent with work, work schedule, legal relation, secondary employment.

Included risk factors and diseases were smoking, alcohol and illicit drug intake, diabetes, hypertension, ischemic heart disease, musculoskeletal pain, history of malignancy and depression.

### 2.2. Psychometric Measures

Internet addiction was detected with the Problematic Internet Use Questionnaire, which is a validated self-report scale with good reliability and validity characteristics [15]. The questionnaire contains 18 items, each scored on a 5-point Likert-type scale ranging from 1 (never) to 5 (always). A confirmatory factor analysis verified the three-factor model of the questionnaire, each subscale containing six items. A total score exceeding 41 points suggests internet addiction [16]. Daily use, time interval and goal of internet use were also recorded. 

Burnout was measured with the Maslach Burnout Inventory [17]. This validated instrument has three subscales and measures emotional exhaustion (being overextended and exhausted by one’s studies), cynicism (distant attitude towards studies) and professional efficacy (satisfaction with past and present accomplishments). Responses are marked on a seven-point Likert scale (0 meaning ‘never’ and 6 meaning ‘every day’) and then summed.

### 2.3. Statistical Analysis

Data were evaluated as means ± SD (standard deviation) by Student’s *t*-test, the chi-square test and the Pearson’s Rank-Order Correlation. Logistic regression analysis was used to determine the significance of the different parameters as independent risk factors of IA. The analysis was performed with appropriate adjustments for differences in risk factors and medication usage. For all odds ratios, an exact CI of 95% was constructed in our study. Data analysis was performed using SPSS (version 22.0, IBM, New York, NY, USA).

## 3. Results

### 3.1. Baseline Characteristics

Overall 600 questionnaires were successfully delivered and 485 responses received (response rate of 80.8%). A total of 49 doctors (10.1%), 198 nurses (40.9%), 123 medical assistants (25.4%), 73 other healthcare workers (15.1%), and 42 (1.7%) healthcare associated workers (cleaning, laundry, etc.) have completed our survey.

A total of 411 females (84.8%) and 74 males (15.2%) participated in our study. The vast majority of the study’s participants were aged between 36 and55 (291/485, 60.1%), were married (333/485, 58.7%), had two children (165/485, 34.1%), had secondary education (165/485, 34.1%) and had been working for 21–40 years (231/485, 47.6%) mainly in acute (223/485, 45.9%) and outpatient care (138/485, 28.5%). Details can be seen in Table 1.

### 3.2. Previous Diseases and Concomitant Medications

A total of 31.8% (154/485) were regular smokers, 2.9% (14/485) drank alcohol and 1.9% (9/485) took illicit drugs more or less regularly.

A total of 26.4% (128/485) had hypertension, 4.5 % (22/485) had diabetes, 12.4 % had ischemic heart disease (60/485), and 17.5% (90/485) suffered from musculoskeletal pain. A total of 3.3 % (16/485) had a history of malignancy and 2.7% (13/485) a history of mood disorder (Supplementary Table S1).

### 3.3. Internet Use

A total of 34% (165/489) spent less than one hour online and 0.8% (4/485) used the internet more than six hours a day. More than half of the examined workers preferred being online between 6 and 9 p.m. (52%, 252/485). The main goals of internet surfing were to visit social media sites in 48.9% (237/485), everyday work in 43.1% (209/485) and listening to music in 37.7% (183/485). Detailed data can be seen in Supplementary Table S1.

### 3.4. Internet Addiction

Internet addiction was detected in 3.9% (19/485) based on the Problematic Internet Use Questionnaire.

### 3.5. Burnout

A total of 24.1% (117/485) suffered from mild, 71.4% (346/485) from moderate and 4.5% (22/485) from severe burnout based on the Maslach Burnout Inventory. Mean values were the following in the subcategories: emotional exhaustion 25.91 ± 9.4 points, depersonalization 19.67 ± 6.7 points, personal accomplishment 9.37 ± 5.1 points. Mild emotional exhaustion occurred in 25.6% as moderate, in 58.7% as severe, and in 15.7% of all workers. The rate of mild, moderate and severe depersonalization was 61.6%, 34% and 4.4%, respectively. A total of 16.2% suffered from mild, 78% from moderate and 5.8% from severe personal accomplishment (not shown).

### 3.6. Risk Factors of Internet Addiction

There was a significant association between the duration of being online and being addicted to the internet (r = 0.46, *p* < 0.001). The cut-off of spending 5 h or more online predicted IA. Being online between 12 and 3 p.m. (3.9 vs. 10.5%) and 3 and 6 p.m. (14.6 vs. 31.6 %, *p* <0.05 in both cases) also predicted IA (Supplementary Table S2). Among the types of internet services, chatting (27.9 vs. 57.9%, *p* = 0.004) and watching movies (28.3 vs. 57.9%, *p* = 0.005) were significantly associated with IA (Supplementary Table S2).

IA was more common in males (26.3 vs. 14.8%, *p* = 0.04) and workers aged between 18 and 25 (42 vs. 6.2%, *p* = 0.047). IA was more prevalent among singles (16.1 vs. 31.6%, *p* < 0.001), unmarried couples (20.4 vs. 36.8%, *p* < 0.001) and childless (26.8 vs. 47.3 %, *p* = 0.049) (Table 2).

Internet addiction was more common among medical clerks (6.2 vs. 21% *p* < 0.001), and among healthcare associated workers (14.8 vs. 21% *p* <0.001) Internet addiction was more common among those working in chronic care (5.8 vs. 15.8%, *p* = 0.011) (Table 2).

Internet addiction was more prevalent among illicit drug users (15.8 vs. 1.3 %, *p* < 0.001) and among diabetic individuals (10.5 vs. 4.8%, *p* = 0.011) (Supplementary Table S2).

### 3.7. Burnout and Internet Addiction

There was no significant association of burnout and internet addiction, also taking the subcategories into account (Table 3).

### 3.8. Multivariate Analysis

In a multivariate analysis including all factors (demographic criteria, burnout, internet habits, comorbidity, etc.), internet addiction was significantly associated with ages 18-25 (OR: 2.6, *p* = 0.024), surfing on the internet > 5 h daily (OR 25.583, *p* < 0.001), being single (OR:4.275, *p* = 0.006), being childless (OR: 3.81, *p* = 0.011), working less than five years (OR 2.135, *p* = 0.048) and job type (being healthcare associated worker, OR: 2.907, *p* = 0.009). Illicit drug intake (OR 52.494, *p* < 0.001) and diabetes (OR: 4.122, *p* = 0.043) were also strongly correlated with IA.

## 4. Discussion

Internet addiction is a well-known phenomenon among adolescents, but only a few studies have focused on its prevalence and risk factors among adults, especially among middle-aged or older people.

IA has been under considerable research, and has generated controversy, debate and quarreling among expert researchers, healthcare and non-healthcare professionals due to insufficient data, poor quality research and lack of randomized studies [18]. However, internet addiction seems to be more than just the consequence of mental instability of adolescents. It can be associated with atrophy in the prefrontal and striatal areas similar to other types of addictions [18]. The key point is the activation of the forebrain dopamine systems. Excessive activation of this system strengthens the specific habits that precede the activation, and overactivation also downregulates the dopamine receptors at the same time, leaving the subject less interested in other activities [19]. In the long term it leads to the malfunction of brain networks necessary for self-regulation, thereby facilitating impulsive, inflexible and compulsive actions [19]. 

IA is associated with substance abuse such as alcohol or drugs and psychiatric symptoms such as depression, sleep disturbance and depression based on recent studies. The association of IA and psychiatric symptoms is not well understood, and may share similar neural circuits including the reward circuits (see above), memory and learning circuits, cognitive control loops [19,20,21]. 

Both sedentary lifestyle and postural habits/long-lasting fixed positions can play a role in the development of musculoskeletal pain, which occurs more frequently among internet addicts [22].

In a recent meta-analysis, the rate of internet addiction was 9.7% among healthcare professionals, which is far lower than it would be expected by the IA rate of medical students, which can be as high as 30% [1,23]. In our study the rate of IA was 3.9%, which is significantly lower compared to the above-mentioned findings, and furthermore, no IA could be found among doctors, which underlies the possible protective effect of growing age (and workload) or late-onset internet use [24].

Problematic internet use was more common among males, younger people (<25 years), singles and childless couples, similar to previous results [25,26,27,28]. These were independent predictors of problematic internet use. We have also found different patterns of internet use as predictors of addiction such as chatting or watching movies, and significant association of the duration of being online and problematic internet use as described before [29,30,31]. Surfing the internet > 5 h daily was a significant predictor of addiction.

IA was more prevalent in medical clerks and among healthcare associated workers, which was not published before. In contrast to medical management of patients, they are involved in electronic health record use, which was recently shown as a contributor to lower work satisfaction and higher frustration, possibly leading to addictive behaviors such as problematic internet use [32]. Secondly, their work is usually computer (internet)-based, and they can easily be online during working hours. The above-mentioned parameters were also significant predictors of IA in a multivariate analysis. 

IA was also more prevalent among those working in chronic care. We have found no explanation for this phenomenon as we found no studies focusing on the association of internet addiction and workflow among healthcare professionals. Possible differences in working conditions (workload, daily working hours, number of patients seen, night duties, sleep irregularity) can explain this finding [33].

Based on a recent meta-analysis each additional 1 h/d of internet use was associated with 8% increased odds of weight gain/excessive weight and obesity, which can probably lead to metabolic syndrome, diabetes and cardiovascular morbidity [20]. IA was a significant predictor of diabetes in this study and our paper is the first showing IA as a possible risk factor of diabetes.

IA was also associated with illicit drug intake both in uni- and multivariate analysis. The pathophysiology is not well-understood, an underlying psychopathology (history of addiction) may precipitate internet addiction or IA may lead to co-addictions, and finally they may enhance each other [33]. 

Recent studies have shown possible association of burnout with physical, psychological and occupational consequences such as cardiovascular disorders, diabetes, depression and addictive behaviour [10,11,12]. There are limited data about the association of burnout and problematic internet use as a behavioural addiction. 

Taking the subcategories of burnout into account, emotional exhaustion can result in higher anxiety levels and reduced communications skills with subsequent social isolation, and can be associated with IA as well as depersonalization, which results in weaker communication or social skills according to a very recent study [34]. However, the causality is not entirely clarified, and only one study has focused on the association of burnout and IA showing positive correlations among healthcare professionals [28].

Although the vast majority of the participants suffered from mild, and 5% from severe burnout, we found no association with IA. This should be interpreted with caution as only half of the participants work in acute patient care, and the number of doctors was pretty low (~10%). The possible association merits further investigation.

In summary, this is the first study from Hungary showing the prevalence and risk factors of internet addiction in a single hospital. A small but significant proportion of our healthcare workers suffered from IA, which was associated with substance abuse and diabetes in a multivariate analysis. Our study also draws attention to the risk factors of IA such as younger age, family status, working type and working hours internet use.

Our study has had some limitations. The study sample was limited and represented individuals working at a single hospital in Hungary. As it was a questionnaire based survey, physical examination was not carried out and we had no detailed information about the medical history of the participants. Finally, follow-up was not carried out.

## 5. Declarations

Ethics approval and consent to participate: The study protocol conforms to the ethical guidelines of the 1975 Declaration of Helsinki as reflected in a priori approval by the Regional Ethical Committee at Szent Rókus Hospital, Baja, Hungary, as seen above.

## Figures and Tables

**Table 1:** Baseline characteristics of the study population.

(*N* = 485)	%
**Gender**	
Female	84.8
Male	15.6
**Age**	
18–25 years	7.7
26–35 years	16.2
36–45 years	29.9
46–55 years	30.2
56–62 years	13.7
more than 62 years	2.3
**Marital status**	
single	16.7
relationship	21.0
married	47.7
divorced/widow	14.6
**Number of children**	
have no child	27.7
1 child	24.8
2 children	34.1
more than 3 children	13.4
**Type of work**	
medical clerk	6.8
assistant	25.4
nurse	40.9
doctor	10.1
other health care worker	15.1
other	1.7
**Years spent with work**	
1–12 months	5.0
1–5 years	13.6
6–10 years	11.1
11–20 years	20.4
21–30 years	25.8
31–40 years	21.8
more than 40 years	2.3
**Workflow**	
acute care	45.9
chronic care	6.1
rehabilitation	8.0
outpatient care	28.5
**Secondary employment**	
yes	82.3
no	17.7

**Table 2:** Comparison of baseline characteristics of the study subgroups.

	Not Addicted to Internet (*n* = 466)	Internet Addiction (*n* = 19)
**Gender**		
Male	**69 (14.8%)**	5 (26.3%) *
Female	397 (85.2%)	14 (73.7%)
**Age (years)**		
18–25 years	**29 (6.2%)**	8 (42%) *
26–35 years	76 (16.3%)	3 (15.8%)
36–45 years	141 (30.2%)	4 (21%)
46–55 years	144 (30.9%)	2 (10.5%)
56–62 years	64 (13.2%)	2 (10.5%)
more than 62 years	12 (2.6%)	0 (0.0%)
**Marital status (%)**		
single	**75 (16.1%)**	6 (31.6%) **
relationship	**95 (20.4%)**	7 (36.8%) **
married	227 (48.7%)	4 (21%)
divorced / widow	69 (14.8%)	2 (10.5%)
**Number of children**		
have no child	**125 (26.8%)**	9 (47.3%) *
1 child	117 (25.1%)	4 (21%)
2 children	163 (35%)	4 (21%)
more than 3 children	63 (13.5%)	2 (10.5%)
**Type of work**		
medical clerk	**29 (6.2%)**	4 (21%) **
assistant	121 (26%)	2 (10.5%)
nurse	190 (40.8%)	8 (42.1%)
doctor	49 (10.5%)	0 (0.0%)
other healthcare worker	69 (14.8%)	4 (21%)
other	8 (1.7%)	1 (5.2%)
**Years spent with work**		
1–12 months	22 (4.7%)	2 (10.5%)
1–5 years	**59 (12.6%)**	7 (36.8%) *
6–10 years	50 (10.7%)	4 (21%)
11–20 years	97 (20.8%)	2 (010.5%)
21–30 years	124 (26.6%)	1 (5.3%)
31–40 years	103 (22.1%)	3 (15.8%)
more than 40 years	11 (2.4%)	0 (0.0%)
**Workflow**		
acute care	216 (46.3%)	7 (36.8%)
chronic care	**27 (5.8%)**	3 (15.8%) *
rehabilitation	37 (7.9%)	2 (10.5%)
outpatient care	136 (29.2%)	2 (10.5%)
healthcare associated work	50 (10.7%)	5 (26.3%)

** *p* < 0.001, * *p* < 0.005.

**Table 3:** Burnout and internet addiction.

	Not Addicted to Internet (*n* = 466)	Internet Addiction (*n* = 19)
Burnout		
low	114 (24.5%)	3 (15.8%)
moderate	330 (70.8%)	16 (84.2%)
severe	22 (4.7)	0 (0.0%)
emotional exhaustion	20.89 ± 9.7	22.9 ± 8.05
depersonalisation	9.32 ± 5.08	9.89 ± 1.13
personal accomplishment	19.53 ± 7.08	19.68 ± 1.9

## Data Availability

The dataset supporting the conclusions of this article is available on request to the corresponding author.

## Supplementary Materials

The following are available online at https://www.mdpi.com/1660-4601/18/2/615/s1.
