# Supplementary material for: Internet Addiction and Burnout in A Single Hospital: Is There Any Association?
Source: Int J Environ Res Public Health. 2021 Jan 13;18(2):615. doi: 10.3390/ijerph18020615 (PMC7828215; doi:10.3390/ijerph18020615)
Supplement: Supplementary file 1 [file ijerph-18-00615-s001.pdf]

International Journal of
Environmental Research and Public Health

MDPI

Supplementary

Table 1. Concomittant diseases. substance abuse and internet use in the study population.

**Concomittant diseases (%)**	
taking any medication regularly	37.3
smoker	31.8
taking alcohol	2.9
taking drugs	1.9
diabetes	4.5
hypertension	26.4
cardiovascular disease	12.4
musculosceletal pain	17.5
history of cancer	3.3
depression or other psychiatric disease	2.7
**Daily internet use (approximately) (%)**	
1 hour	34.0
2 hours	26.8
3 hours	22.7
4 hours	7.4
5 hours	4.3
6 hours	4.0
> 6 hours	0.8
**Daily time interval of internet use (multiply answer) (%)**	
between 0-3 am.	16.1
between 3-6 am.	2.5
between 6-9 am.	5.2
between 9-12 am.	8.5
12-3 pm	4.1
3-6 pm.	15.3
6-9 pm.	52.0
9.12 pm.	13.2
**Goal of internet use (multiply answer) (%)**	
learning/working	43.1
internet gaming	11.3
chat	29.1
community portal (Facebook. Twitter etc)	48.9
matchmaking	1.2
movies	29.5
music	37.7
other	22.3

Table 2. Comparism of concomittant diseases. substance abuse and internet use in the study subgroups.

	Not addicted to internet (n= 466)	Internet addiction (n= 19)
**Concomittant diseases**		
taking any medication regularly	175 (37.6%)	6 (31.6%)
smoker	145 (31.1%)	9 (47.3%)
taking alcohol	13 (2.8%)	1 (5.3%)
**taking drugs**	**6 (1.3%)**	**3 (15.8%)****
**diabetes**	**19 (4.8%)**	**2 (10.5%)***
hypertension	121 (26%)	7 (36.8%)
cardiovascular disease	55 (11.8%)	5 (26.3%)
musculosceletal pain	81 (17.4%)	4 (21%)
history of cancer	15 (3.2%)	1 (5.2%)
depression or other psychiatric disease	12 (2.6%)	1 (5.2%)
**Daily internet use (approximately)**		
1 hour	163 (35%)	2 (10.5%)
2 hours	127 (27.2%)	3 (15.8%)
3 hours	102 (21.9%)	8 (42.1%)
4 hours	33 (7.1%)	3 (15.8%)
**5 hours**	**19 (4.1%)**	**2 (10.5%)****
6 hours	18 (3.9%)	1 (5.2%)
> 6 hours	4 (0,8%)	0 (0,0%)
**Daily time interval of internet use (multiply answer)**		
between 0-3 am.	77 (16.5%)	1 (5.2%)
between 3-6 am.	10 (2.1%)	2 (10.5%)
between 6-9 am.	24 (5.2%)	1 (5.2%)
between 9-12 am.	39 (8.4%)	2 (10.5%)
**12-3 pm**	**18 (3.9%)**	**2 (10.5%)***
**3-6 pm.**	**68 (14.6%)**	**6 (31.6%)***
6-9 pm.	242 (52%)	10 (52.6%)
9.12 pm.	64 (13.7%)	0 (0,0%)
**Goal of internet use (multiply answer)**		
learning/working	201 (43.1%)	8 (42.1%)
internet gaming	51 (10.9%)	4 (21%)
**chat**	**130 (27.9%)**	**11 (57.9%)***
community portal (Facebook. Twitter etc)	225 (48.3%)	12 (63.2%)
matchmaking	5 (1.0%)	1 (5.2%)
**movies**	**132 (28.3%)**	**11 (57.9%)***
music	173 (37.1%)	10 (52.6%)
other	100 (21.5%)	3 (15.8%)

$**p<0.001$

$*p<0.05$
